# Stress-Induced Changes of Hippocampal NMDA Receptors: Modulation by Duloxetine Treatment

**DOI:** 10.1371/journal.pone.0037916

**Published:** 2012-05-29

**Authors:** Francesca Calabrese, Gianluigi Guidotti, Raffaella Molteni, Giorgio Racagni, Michele Mancini, Marco Andrea Riva

**Affiliations:** 1 Center of Neuropharmacology, Department of Pharmacological and Biomolecular Sciences, Università degli Studi di Milano, Milan, Italy; 2 Medical Department, Eli Lilly Italia S.p.A., Sesto Fiorentino, Italy; 3 Center of Excellence on Neurodegenerative Diseases, Università degli Studi di Milano, Milan, Italy; University of Texas Health Science Center at San Antonio, United States of America

## Abstract

It is now well established that the glutamatergic system contributes to the pathophysiology of depression. Exposure to stress, a major precipitating factor for depression, enhances glutamate release that can contribute to structural abnormalities observed in the brain of depressed subjects. On the other hand, it has been demonstrated that NMDA antagonists, like ketamine, exert an antidepressant effect at preclinical and clinical levels. On these bases, the purpose of our study was to investigate whether chronic mild stress is associated with specific alterations of the NMDA receptor complex, in adult rats, and to establish whether concomitant antidepressant treatment could normalize such deficits. We found that chronic stress increases the expression of the obligatory GluN1 subunit, as well as of the accessory subunits GluN2A and GluN2B at transcriptional and translational levels, particularly in the ventral hippocampus. Concomitant treatment with the antidepressant duloxetine was able to normalize the increase of glutamatergic receptor subunit expression, and correct the changes in receptor phosphorylation produced by stress exposure. Our data suggest that prolonged stress, a condition that has etiologic relevance for depression, may enhance glutamate activity through post-synaptic mechanisms, by regulating NMDA receptors, and that antidepressants may in part normalize such changes. Our results provide support to the notion that antidepressants may exert their activity in the long-term also via modulation of the glutamatergic synapse.

## Introduction

Major depression is a serious, debilitating, life-shortening illness affecting millions of people worldwide, which arises from the complex interaction between susceptibility genes and environmental factors, such as stress [1]. Available drugs, which have been used for more than 50 years, are based on the increase of biogenic amines at synaptic level. While these treatments require 2–4 weeks to produce a clinically meaningful improvement, only 60–65% of patients respond to the initial regimen and, among these, less than half reach remission or become symptom-free [2].

A major obstacle to the development of more effective treatments for major depression has been the limited understanding of its pathophysiology, and of the mechanisms that may be relevant for clinical efficacy. Within this context, dysfunction of the glutamatergic system has emerged as a major pathological feature in depression, and may represent a target for pharmacological intervention [3].

Genetic evidence for the involvement of the glutamatergic pathway in emotional regulation has been obtained by analyzing mice with genetically engineered NMDA receptor genes [4], [5]. Early studies also showed that acute restraint stress induces an increase of extracellular levels of excitatory neurotransmitters (glutamate and aspartate) in different brain structures [6], and, more recently, that chronic stress causes alterations of the glutamatergic system, which may lead to dendrite retraction in hippocampal subfields [7], [8]. Moreover, animals which undergo chronic restraint stress paradigms show an increase in basal, as well as in depolarization-dependent, glutamate release from hippocampal synaptosomes, suggesting a dysregulation in the mechanism responsible for termination of glutamate secretion [9].

Based on these premises, several glutamatergic modulating agents have been used to revert the behavioural and molecular alterations present in mood disorders. The hypothesis that NMDA antagonists have antidepressant properties was first proven after the examination of several glutamatergic modulators in a murine variant of the forced swim test [10]. At clinical level, a subanesthetic dose of ketamine, a non-selective NMDA receptor antagonist, has been shown to induce rapid antidepressant effects that are sustained for several days after a single infusion [11], [12]. Ketamine has also been consistently shown to possess antidepressant-like properties in different rodent models of depression [13], [14], [15].

Since stress-induced enhancement of glutamate release and transmission is a crucial factor for the induction of structural and functional changes associated with depression, and knowing that glutamate modulators show antidepressant activity, it would be interesting to investigate whether currently used antidepressants are able to interfere with stress-induced alterations of glutamate transmission. However, this aspect has been poorly addressed. There is, for example, evidence that antidepressant drugs can prevent the enhancement of depolarization-dependent glutamate release induced in the rat prefrontal/frontal cortex by acute footshock-stress [16].

NMDA receptors are tetrameric ion channels comprising the obligatory subunit GluN1 and the modulatory subunits NR2 (A–D) and NR3 (A and B) [17], [18]. The GluN2 subunits substantially contribute to functional and anatomical diversities of NMDA receptors. In particular, within in the adult brain, the NR2A and NR2B subunits are highly expressed in the in structures that are important for mood disorders. On these bases, understanding the regulation of NR2 subunits in pathological conditions is important to develop subunit-selective strategies aimed at a pharmacological modulation of glutamate dysfunction [19].

On this basis, the specific aim of this study was to characterize the alterations of the NMDA receptor complex in the chronic mild stress (CMS) model of depression, and to establish the impact of chronic antidepressant treatment on the observed changes. We decided to investigate hippocampus and prefrontal cortex, two key regions for mood disorders and stress-related pathologies. Moreover, with respect to the hippocampus we separately investigated the ventral subregion, which is implicated in anxiety-related behaviors, and dorsal part that has a preferential role in spatial learning and memory [20], [21].

## Materials and Methods

### Ethics Statement

All animal handling and experimental procedures were performed in accordance with the EC (EEC Council Directive 86/609 1987), the Italian legislation on animal experimentation (Decreto Legislativo 116/92), and the National Institutes of Health Guide for the Care and Use of Laboratory Animals. All efforts were made to minimize animal suffering and to reduce the number of animals used. We obtained the ethics approval for this study from the Italian Ministry of Public health, Department of veterinary public health, approval ID 475.

### Animals

Adult (3 months old) male Sprague-Dawley rats (Charles River, Calco, Italy), weighing 225–250 g at the start of the experiment, were housed in groups of 3 per cage under standard conditions (12-h light/dark cycle, light off at 7 p.m.) and were exposed to daily handling for 2 weeks before any procedure.

### Reagents

Reagents were purchased from Sigma–Aldrich (Milan, Italy), Bio-Rad Laboratories S.r.l. Italia (Segrate, Italy), Eurofins MWG-Operon, (Ebersberg, Germany), Tebu-bio (Magenta, Italy), GE Healthcare Europe GmbH (Pero, Italy).

### Chronic Stress Paradigms and Antidepressant Treatment

For chronic mild stress (CMS), animals were exposed for 6 weeks to a variable sequence of mild, unpredictable stressors, manipulation that leads to a chronic depressive-like state that develops gradually over time. The animals were exposed once or twice daily to different stressors including food or water deprivation, crowding, isolation, soiled caged, 2 h immobilization, light on overnight. Controls animals were handled every 2 days during weighing. In order to evaluate the amount of food eaten and water drunk, every morning both experimental groups were weighed and refilled to avoid the possibility that animals remained without pellet and/or water. For antidepressant treatment animals were or were not (stress or no-stress) exposed to CMS for 21 days and then administered once a day with duloxetine, a serotonin and noradrenaline reuptake inhibitor (SNRI) [22] (10 mg/kg, per os, by gavage), or vehicle (hydroxyethylcellulose, HEC, 1%, 1 ml/kg, per os, by gavage) with continued CMS (total of 42 days).

On day 43, in the morning, rats were killed by decapitation 24 h after the last duloxetine administration. The hippocampus (dorsal and ventral) and the prefrontal cortex were rapidly dissected. The dorsal hippocampus corresponds to the plates 25–33 according to the atlas of Paxinos and Watson (Paxinos and Watson 1996), whereas the ventral hippocampus corresponds to the plates 34–43. The prefrontal cortex (defined as Cg1, Cg3, and IL subregions corresponding to the plates 6–10 according to the atlas of Paxinos and Watson) was dissected from 2-mm-thick slices, whereas the hippocampus was dissected from the whole brain. The brain specimens were frozen on dry ice and stored at −80°C for further analysis.

### RNA Preparation and Gene Expression Analysis by Quantitative Real-time PCR

Total RNA was isolated by single step of guanidinium isothiocyanate/phenol extraction using PureZol RNA isolation reagent (Bio-Rad Laboratories s.r.l. Italia) according with the manufacturer’s instructions and quantified by spectrophotometric analysis. Following total RNA extraction, the samples were processed for real-time polymerase chain reaction ***(RT-PCR)*** to assess GluN1, Glun2A and GluN2B mRNA levels. An aliquot of each sample was treated with DNase to avoid DNA contamination. RNA was analyzed by TaqMan qRT-PCR instrument (CFX384 real time system, Bio-Rad Laboratories, Italy) using the iScriptTM one-step RT-PCR kit for probes (Bio-Rad Laboratories). Samples were run in 384 well formats in triplicate as multiplexed reactions with a normalizing internal control (36B4). Primer sequences used (Table 1) were purchased from Eurofins MWG-Operon.

**Table 1:** Sequences of forward and reverse primers used in real time PCR analysis.

	FORWARD primer	REVERSE primer
GluN1	TCATCTCTAGCCAGGTCTACG	CAGAGTAGATGGACATTCGGG
GluN2A	GCACCAGTACATGACCAGATTC	ACCAGTTTACAGCCTTCATCC
GluN2B	TTCATGGGTGTCTGTTCTGG	GGATGTTGGAGTGGGTGTTG
36B4	TTCCCACTGGCTGAAAAGGT	CGCAGCCGCAAATGC

Thermal cycling was initiated with an incubation at 50°C for 10 min (RNA retrotranscription) and then at 95°C for 5 min (TaqMan polymerase activation). After this initial step, 39 cycles of PCR were performed. Each PCR cycle consisted of heating the samples at 95°C for 10 s to enable the melting process and then for 30 s at 60°C for the annealing and extension reactions. A comparative cycle threshold (Ct) method was used to calculate the relative target gene expression.

### Analysis of NMDA Subunit Protein Levels

Western blot analysis was used to investigate NMDA subunit protein levels in the crude synaptosomal fraction. Tissues were manually homogenized using a glass-glass potter in a pH 7.4 cold buffer containing 0.32 M sucrose, 0.1 mM EGTA, 1 mM HEPES solution in presence of a complete set of protease (Roche) and phosphatase (Sigma-Aldrich) inhibitors. The total homogenate was centrifuged at 2,500 rpm for 10 min at 4°C. The supernatant obtained was centrifuged at 10,000 g for 15 min at 4°C to obtain a pellet corresponding to the crude synaptosomal fraction which was re-suspended in a buffer (20 mM HEPES, 0.1 mM dithiothreitol (DTT), 0.1 mM EGTA) with protease and phosphatase inhibitors. Total protein content was measured according to the Bradford Protein Assay procedure (Bio-Rad Laboratories), using bovine serum albumin as calibration standard.

Equal amounts of protein were run under reducing conditions on 10% SDS-polyacrylamide gels and then electrophoretically transferred onto nitrocellulose membranes (Bio-Rad Laboratories). The blots were blocked with 10% nonfat dry milk and then incubated with the primary antibodies summarized in the Table 2. Membranes were then incubated for 1 h at room temperature with the opportune secondary antibody (see Table 2), and immunocomplexes were visualized by chemiluminescence using the ECL Western Blotting kit (GE Healthcare Europe GmbH).

**Table 2:** Conditions of the antibodies used in the Western blot analysis.

	Primary antibody	Secondary antibody
Phospho GluN1 (Ser 896)	1∶1000 (Santa Cruz), 4°C, O/N	anti-rabbit, 1∶5000, RT, 1 h
Phospho GluN1 (Ser 897)	1∶2500 (Cell Signaling), 4°C, O/N	anti-rabbit, 1∶5000, RT, 1 h
Phospho GluN2B (Ser 1303)	1∶1000 (Upstate), 4°C, O/N	anti-rabbit, 1∶2000, RT, 1 h
Phospho GluN2B (Tyr 1472)	1∶1000 (Millipore), 4°C, O/N	anti-rabbit, 1∶2000, RT, 1 h
Total GluN1	1∶1000 (Zymed), 4°C, O/N	anti-mouse, 1∶3000, RT, 1 h
Total GluN2A	1∶1000 (Zymed), 4°C, O/N	anti-mouse, 1∶2000, RT, 1 h
Total GluN2B	1∶1000 (Santa Cruz), 4°C, O/N	anti-goat, 1∶2000, RT, 1 h
β-actin	1∶10000 (Sigma), 4°C, O/N	anti-mouse, 1∶10000, RT, 1 h

Results were standardized using β-actin as the control protein, which was detected by evaluating the band density at 43 kDa. Protein levels were calculated by measuring the optical density of the autoradiographic bands using Quantity One software (Bio-Rad Laboratories). To ensure that autoradiographic bands were in the linear range of intensity, different exposure times were used.

### Statistical Analyses

The effects of stress and/or antidepressant treatment were analyzed with a two-way analysis of variance (ANOVA) followed by Single Contrast Post Hoc Test (SCPHT). Significance for all tests was assumed for *p*<0.05. Data are presented as means ± standard error (SEM). For graphic clarity, results are presented as mean percent of No Stress/Vehicle-treated rats.

## Results

### Modulation of Glutamate NMDA Receptor Subunit Expression in CMS Rats

The purpose of this study was dual. On one hand we want to investigate the effect of chronic mild stress on different component of the glutamatergic system, on the other we examined the impact of chronic antidepressant treatment on the changes produced by prolonged stress exposure. To this purpose, the SNRI duloxetine was given for 3 weeks to either sham or CMS rats, starting from the end of the third week of stress.

**Figure 1:**
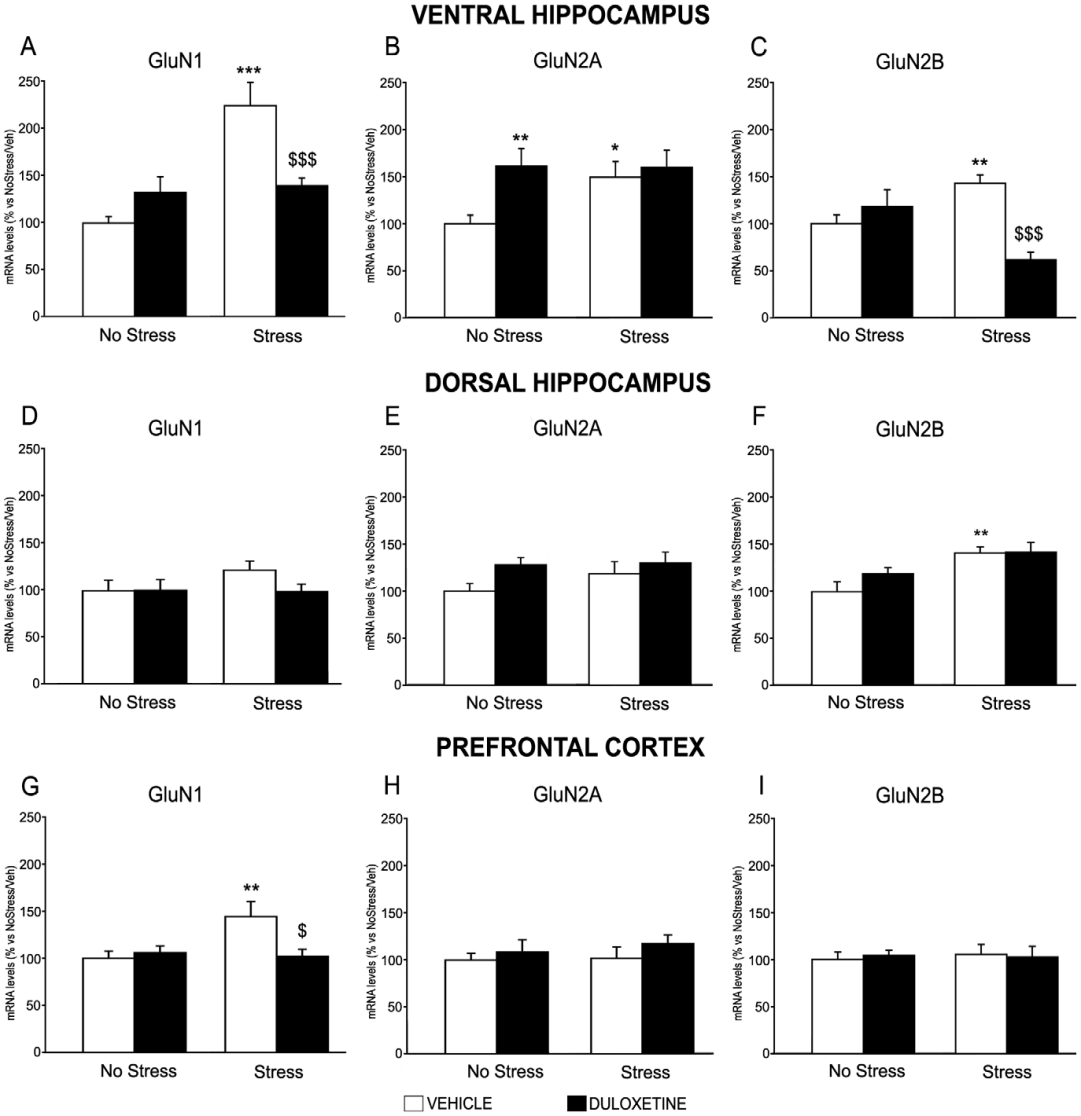
Modulation of NMDA receptor subunits expression by chronic stress and antidepressant treatment *in rat brain.* The mRNA levels of GluN1 *(* ***A, D, G*** *),* GluN2A *(* ***B, E, H*** *)* and GuN2B *(* ***C, F, I*** *)* were measured in ventral hippocampus *(* ***A, B, C*** *),* ***dorsal hippocampus*** *(* ***D, E, F*** *)* ***and prefrontal cortex*** *(* ***G, H, I*** *)* of non stressed and chronically stressed rats, treated for 21 days with vehicle or duloxetine and killed 24 hours after the last stress. The data, expressed as a percentage of No Stress/Vehicle (set at 100%), are the mean ± SEM of at least 10–12 independent determinations. *p<0.05, **p<0.01, ***p<0.01 vs. No Stress/Vehicle; *^$^* ***p<0.05***, ^$$$^p<0.001 vs. Stress/Vehicle (two-way ANOVA with SCPHT).

As shown in Fig. 1A, in ventral hippocampus we found a significant effect of stress (F_1,47_ = 16.186, p<0.001) on GluN1 expression as well as a significant stress X drug interaction (F_1,47_ = 12.790, p<0.001): indeed, the mRNA levels for the key NMDA receptor subunit were significantly increased following CMS in vehicle-treated rats (+125*±25*%, p<0.001 vs. No Stress animals), an effect that was significantly reduced in rats chronically administered with duloxetine (−38*±8*%, p<0.001 vs. Stress animals). A similar pattern of changes was also found with respect to GluN2B, with a statistically significant stress X drug interaction (F_1,46_ = 23.332, p<0.001). In fact, duloxetine treatment, which did not alter GluN2B mRNA levels in sham animals, was able to normalize (−51*±7*%, p<0.001 vs Stress animals) the increased expression found in CMS rats (+141*±7*%, p<0.001 vs No Stress animals) (Fig. 1C).

Conversely, the regulation of GluN2A did not undergo similar changes. In fact, the significant stress X drug interaction (F_1,45_ = 4.260, p<0.05) was due to the fact that the antidepressant increased the mRNA levels of GluN2A in sham animals (+62*±15*%, p<0.001 vs No Stress animals), but it was not able to counteract the enhanced mRNA levels for this subunit (+52*±13*%, p<0.001 vs Stress animals) when given to CMS rats (Fig. 1B).

In dorsal hippocampus (Fig.1D–F) we found only a significant effect of the chronic stress only regard to the gene expression of GluN2B (Fig. 1F) (F_1,42_ = 13.325, p<0.001). In fact, CMS increased the levels of this specific subunit (+41*±6*% p<0.01 vs. No Stress animals), while duloxetine did not exert any effects.

On the contrary, in prefrontal cortex (Fig. 1G–I), we observed only a significant stress X drug interaction (F_1,43_ = 4.995, p<0.05). Specifically chronic stress significantly increased GluN1 mRNA levels (+44*±16*% p<0.01 vs. No Stress animals), effects corrected by the duloxetine treatment (−30*±7*%, p<0.05 vs Stress animals).

### Modulation of Glutamate NMDA Receptor Subunit Protein Levels and Phosphorylation in CMS Rats

**Figure 2:**
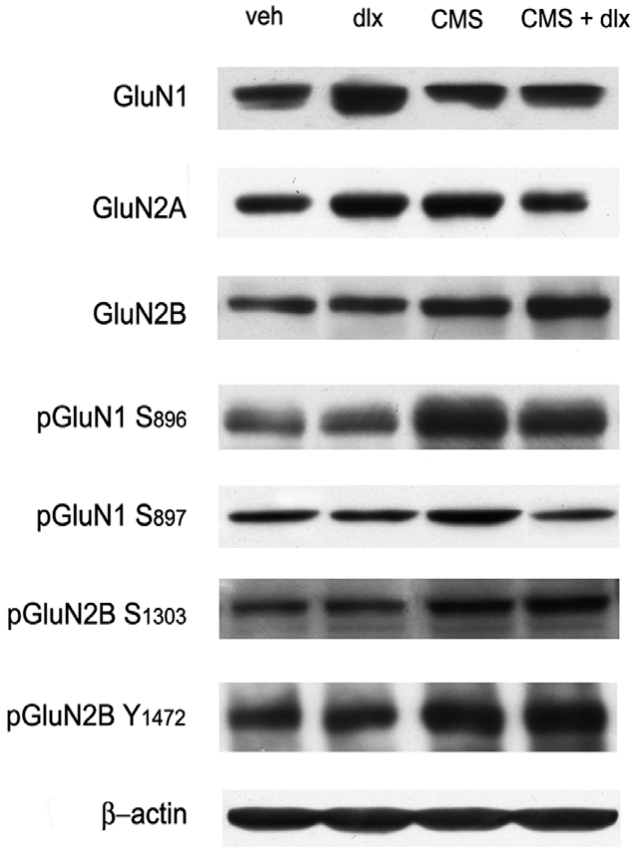
Representative Western blotting of NMDA receptor subunits in ventral hippocampus. Representative western blot analysis of of GluN1, GluN2A, GluN2B, phospho GluN1 (Ser896), phospho GluN1 (ser897), phospho GluN2B (Ser1303), phospho GluN2B (Tyr1472) expression in crude synaptosomal fraction from adult brain ventral hippocampus. β-actin is shown as control for comparison. Veh vehicle, dlx duloxetine, CMS chronic mild stress, CMS + dlx chronic mild stress + duloxetine. Experimental conditions are described in Material and Methods.

We next decided to examine the protein levels of NMDA receptor subunits, specifically in the crude synaptosomal fraction. The analysis was carried out on total protein levels as well as on their phosphorylated forms, a measure that may hold implications with regard to sub-cellular trafficking, activation and synaptic localization [17], [18]. Figure 2 displays prototypical western blot analyses of these proteins. *(* *Fig.2* *)*.

**Figure 3:**
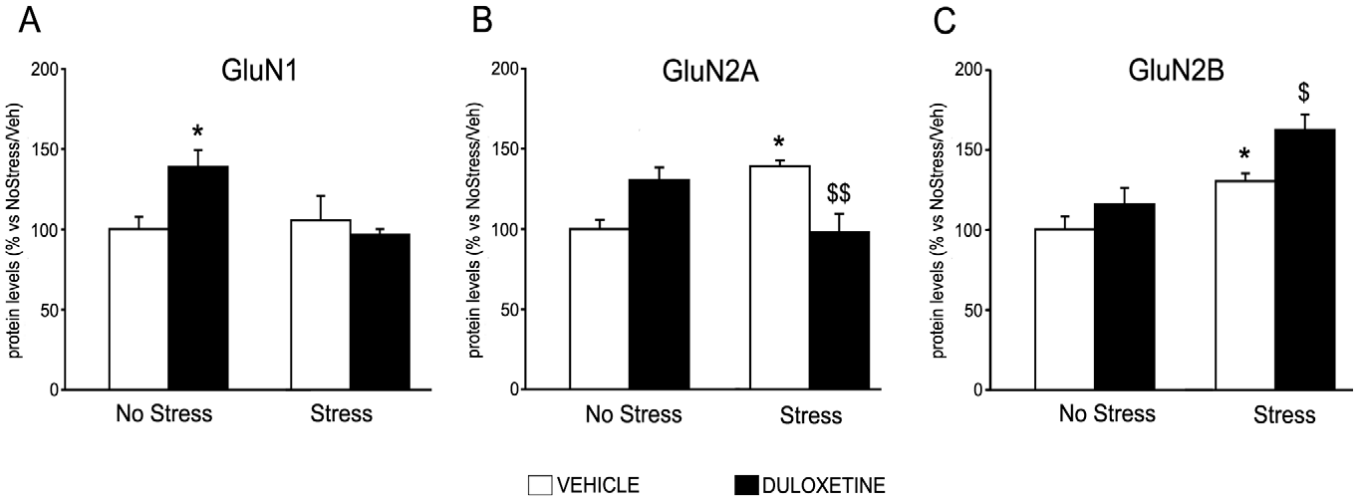
Modulation of protein levels for NMDA receptor subunits by CMS and antidepressant treatment in rat ventral hippocampus. The protein levels of GluN1 (A), GluN2A (B) and GuN2B (C) were measured in the crude synaptosomal fraction of ventral hippocampus of non stressed and chronically stressed rats, treated for 21 days with vehicle or duloxetine and killed 24 hours after the last stress. The data, expressed as a percentage of No Stress/Vehicle (set at 100%), are the mean ± SEM of at least 5–7 independent determinations. *p<0.05 vs. No Stress/Vehicle; ^$^p<0.05, ^$$^p<0.01 vs. Stress/Vehicle (two-way ANOVA with SCPHT).

When investigating the levels of GluN1, we found that the changes in the total receptor subunit protein did not reflect the alterations found at mRNA levels, since CMS did not alter its expression, whereas chronic duloxetine led to a significant increase of GluN1 protein levels in the synaptosomal fraction of sham (+39*±11*%, p<0.05 vs. No Stress animals) but not of CMS exposed rats (−9*±4*%, p>0.05 vs. No Stress animals). The levels of GluN2A (Fig. 3B) mirrored only in part the changes found in gene expression, since, similarly to the modulation of its mRNA levels, CMS produced a significant up-regulation of its protein levels in the synaptosomal fraction (+39*±4*%, p<0.05). Chronic duloxetine completely reversed the up-regulation found in stressed rats, and it produced a slight, although not significant, elevation of receptor subunit expression in control animals (+31*±8*%, p>0.05). With respect to GluN2B (Fig. 3C), we found that, in line with mRNA changes, CMS increased its total protein levels in the synaptosomal compartment, although duloxetine failed to normalize such effect.

**Figure 4:**
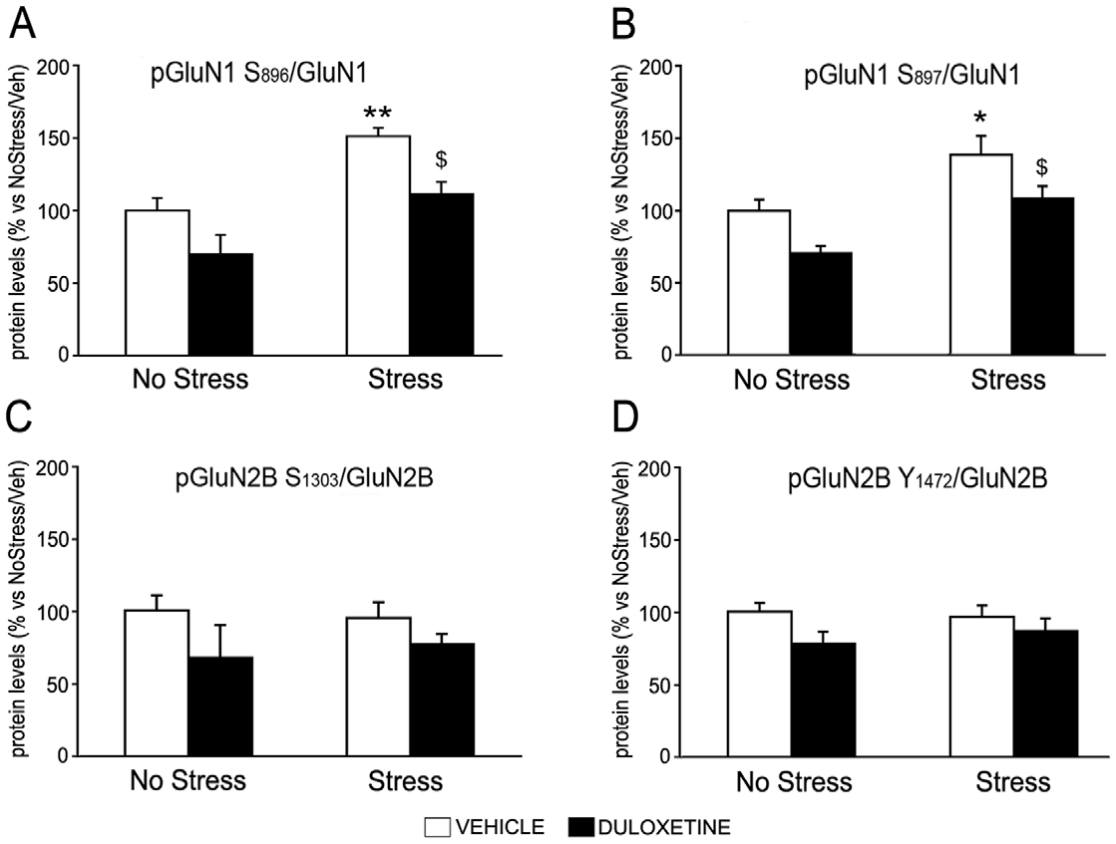
Modulation of GluN1 and GluN2B subunits phosphorylation by CMS and antidepressant treatment in rats ventral hippocampus. The levels of pGluN1Ser896 and pGluN1Ser897 (A,B) or pGluN2BSer1303 and pGluN2BTyr1472 (C,D) were measured in the crude synaptosomal fraction of ventral hippocampus of non stressed and chronically stressed rats, treated for 21 days with vehicle or duloxetine and killed 24 hours after the last stress. The data represent the ration between phosphorylated and total subunit levels. Results, expressed as a percentage of No Stress/Vehicle (set at 100%), are the mean ± SEM of at least 5–7 independent determinations. *p<0.05, **p<0.01 vs. No Stress/Vehicle; ^$^p<0.05 vs. Stress/Vehicle (two-way ANOVA with SCPHT).

Interestingly, when looking at phospho/total GluN1 levels, the pattern was quite similar to the modifications found at mRNA levels (Fig. 4A,B). In particular, for the two phosphorylated sites (pSer896 and pSer897) the ratio phospho/total GluN1 was significantly increased upon exposure to CMS (+51*±6*%, p<0.01 and +39*±13*%, p<0.05, respectively), an effect that was normalized when duloxetine was given during the last three weeks of stress exposure. Exposure to the antidepressant tended to reduce the phospho/total ratio also in sham animals (−30±13% and −30±5% respectively for serine 896 and for serine 897), although the effect did not reach statistical significance.

Furthermore, neither CMS nor the pharmacological treatment was able to alter the degree of GluN2B phosphorylation at two different sites: pSer1303, which is driven by αCaMKII [23], and pTyr1472, that linked to the activation of the fyn pathway [24] (Fig. 4C,D).

## Discussion

The data emerging from the present work provide novel information with respect to changes of glutamate NMDA receptor subunits as a consequence of exposure to CMS, which are to great extent normalized by concomitant treatment with the antidepressant duloxetine. While some of the changes found in the synaptosomal compartment may be sustained by transcriptional mechanisms, there is also evidence that some critical modifications may occur through post-translational modifications, including changes in the phosphorylation of specific receptor subunits, which is well-recognized as being a key process involved in receptor trafficking and synaptic localization [18].

While changes in the mRNA levels of the glutamate NMDA receptor subunits may occur in different brain structures, the major effect take place in the ventral hippocampus, which is strongly relates to stress, emotion, and mood [20].

Although the involvement of glutamate in major depression dates back to several years ago [25], there has been renewed interest in this system because of the observation that NMDA receptor antagonists, such as ketamine, may exert rapid (within 2 hours) antidepressant effects [3], [12]. In particular, pioneer work from Zarate and coworkers has demonstrated that a single intravenous administration of ketamine at a sub-anesthetic dose elicited a rapid improvement of depressive symptoms in resistant patients [12]. It may be inferred that depression-related dysfunctions could be associated with a state of excessive function of the glutamatergic system and/or activation of glutamate receptors, which can be reversed by NMDA receptor blockade [3], [26]. Such view is in accordance with the possibility that excessive NMDA receptor activation represents the point of convergence of different elements that participate to depression susceptibility. One of the main vulnerability factors is stress, which may enhance glutamate release, eventually leading to toxic effects on selected neuronal populations [16], [27]. Our results provide support to this possibility and suggest a novel mechanism that may contribute to the adverse effects of prolonged stress exposure, namely an up-regulation of the NMDA receptor complex occurring at transcriptional level as well as through post-translational mechanisms (receptor phosphorylation at key ***amino acids***) that govern receptor localization and function. This ‘up-regulation’ may eventually amplify glutamatergic signalling, leading to an imbalance of neurotransmitter function, particularly in the ventral hippocampus.

We have previously shown that the phosphorylation of GluN1 on Ser 896 is not affected by acute stress [28], suggesting that the changes observed after CMS may represent a neuroadaptive consequence rather than a rapid modification of receptor activation due to stress exposure. It may be inferred that the glutamatergic system undergoes different changes based on timing and length of the adverse experience. Acute stress can increase the release of glutamate in different brain regions, with a main effect in the prefrontal cortex [6], [16]. This may initiate downstream changes, which could result in coping or susceptibility on the basis of the intracellular pathways that are activated. For example, acute glucocorticoids modulate synaptic transmission and plasticity through AMPA receptors, promoting the consolidation of behavior [29]. On the other hand, chronic stress may lead to a prolonged increase of glutamate levels [30] that, as suggested by the present results, may synergize with a dysfunctional receptor at post-synaptic level.

The transcriptional changes of GluN1 and GluN2B subunits induced by CMS are normalized by chronic exposure to the antidepressant duloxetine, whereas the modulation of GluN2A appears to be somewhat different. Indeed, the significant up-regulation observed in stressed animals is not normalized by antidepressant treatment, whereas chronic duloxetine was able to increase its mRNA levels in control rats.

Previous work has shown that the NMDA receptor complex undergoes changes following chronic treatment with antidepressants in normal animals, which lead to a reduction of its function [10], [31], [32]. Our results show that administration of duloxetine in control animals has limited effect on the expression (mRNA and protein) of NMDA receptor subunits.

One interesting, and previously unexplored, mechanism, is the modulation of the phosphorylation state of the GluN subunits in the synaptosomal compartment. Our results demonstrate that the phosphorylation of GluN1 on serine 896 and 897 is enhanced by stress, which may lead to an increased synaptic localization of the receptor. Indeed, the phosphorylation of GluN1 promotes the exit of the subunit from the endoplasmatic reticulum and increases surface membrane localization [18].

Chronic treatment with the antidepressant duloxetine produced a non significant reduction of GluN1 and GluN2B phosphorylation in normal animals, suggesting that prolonged exposure to antidepressants may indeed lead to a functional inhibition of the receptor complex [18], [31], [32], [33]. More importantly, chronic duloxetine administration led to a significant decrease of GluN1 phosphorylation in stressed rats, with a normalization of the changes observed after exposure to CMS. These results, together with the evidence that mice expressing mutant GluN2A with a Tyr-1325-Phe mutation that prevents its phosphorylation show antidepressant-like behaviour [34], provide support to the notion that NMDA receptor phosphorylation and downstream signalling can modulate depression-related behaviour.

Based on these data, it may be hypothesized that one important adaptive change occurring after long term exposure to duloxetine is a modulation of the GluN subunits transcription and of their localization and activation at synaptic level, which may correct abnormalities produced by a prolonged stress exposure. Interestingly, it has been recently demonstrated that a single dose of ketamine completely reverses the behavioral deficits caused by long-term exposure to CMS, with similar effects occurring also with a selective GluN2B antagonist, Ro 25-6981 [14], [35], [36]. These data suggest that the ‘normalization’ of glutamate-related dysfunctions may indeed represent an important component of the therapeutic response, which may ultimately lead to an improvement of the structural alterations associated with mood dysfunction [3].

It is feasible to hypothesize that, although the effects of classical antidepressants and of ketamine (or glutamate NMDA receptor antagonists) may converge on the same target, the modulation following prolonged exposure to antidepressants is probably part of a complex array of neuroadaptive changes produced by pharmacological intervention [37], [38].

Differently from duloxetine, we have previously shown that sub-chronic electroconvulsive treatment treatment produces a marked increase of the phosphorylation of GluN2B (Ser1303), with no effects in the obligatory subunit GluN1 [39]. Increased phosphorylation of GluN2B on Ser1303 may reduce NMDA receptor activity, presumably uncoupling this receptor subunit from its signalling partner CaMKII [40]. This suggests that antidepressant treatments may differentially regulate the expression and function of NMDA receptor subunits, a common endpoint being the reduction of its functional activity.

Antidepressant activity may therefore originate directly from the reduced function of NMDA receptors, as indicated by our data as well as by previous work that investigated receptor binding and function [15], but it may also be related to increased function of AMPA receptors. Indeed, antidepressant-like effects produced by ketamine treatment and by the GluN2B selective antagonist Ro25-6981 are attenuated by AMPA receptor antagonism. Furthermore, chronic ECT or chronic imipramine treatment lead to enhanced phosphorylation or synaptic localization of GluA1 subunit [39], [41].

Taken together, our results and previous published work suggest that an important mechanism that may contribute to antidepressant activity is the interference with glutamatergic neurotransmission, in order to ‘restore’ altered function in depressive-related conditions. This may indeed occur at several levels that take into account the complex function of this neurotransmitter. Indeed, antidepressants may act at a pre-synaptic level, dampening the excessive release of glutamate [16], and could also interfere with post-synaptic mechanisms, that will globally lead to a reduction of NMDA receptor function and transmission, eventually favoring AMPA receptor transmission [10], [31], [32], [39], [41]. Moreover, antidepressants may also act on glial cells to restore their function, which is crucial for controlling the proper concentration of glutamate at synaptic level [14], [42].

The characterization of these events and of the mechanisms that lie downstream from the glutamatergic synapse will be important to develop novel pharmacological strategies that, alone or in combination with currently used drugs, may improve treatment outcome in depressed patients.
